# Supplementary material for: Potential Distribution of and Sensitivity Analysis for Urochloa panicoides Weed Using Modeling: An Implication of Invasion Risk Analysis for China and Europe
Source: Plants (Basel). 2022 Jul 1;11(13):1761. doi: 10.3390/plants11131761 (PMC9269421; doi:10.3390/plants11131761)
Supplement: Supplementary file 1 [file plants-11-01761-s001.zip › plants-1761802-supplementary.pdf]

Annex I: Occurrences of *Urochloa panicoides* collected in Global Bio-diversity Information Facility (GBIF), Invasive Species Compendium (CABI) and in published literature

ID	Species	Localization	Latitude	Longitude	Registration base	Source
1	*Urochloa panicoides*	New South Wales/ Australia	-35,970	147,000	Human observation	[21]
2	*Urochloa panicoides*	Queensland/ Australia	-27,486	151,779	Human observation	[21]
3	*Urochloa panicoides*	Queensland/ Australia	-27,436	151,701	Human observation	[21]
4	*Urochloa panicoides*	Queensland/ Australia	-27,582	151,994	Human observation	[21]
5	*Urochloa panicoides*	Queensland/ Australia	-27,493	151,795	Human observation	[21]
6	*Urochloa panicoides*	Queensland/ Australia	-27,479	151,757	Human observation	[21]
7	*Urochloa panicoides*	Queensland/ Australia	-27,425	151,692	Human observation	[21]
8	*Urochloa panicoides*	Queensland/ Australia	-27,442	151,706	Human observation	[21]
9	*Urochloa panicoides*	Queensland/ Australia	-27,490	151,790	Human observation	[21]
10	*Urochloa panicoides*	Queensland/ Australia	-27,540	151,983	Human observation	[21]
11	*Urochloa panicoides*	Queensland/ Australia	-27,458	151,724	Human observation	[21]
12	*Urochloa panicoides*	Queensland/ Australia	-27,538	151,979	Human observation	[21]
13	*Urochloa panicoides*	Queensland/ Australia	-27,490	151,789	Human observation	[21]
14	*Urochloa panicoides*	Queensland/ Australia	-27,483	151,769	Human observation	[21]
15	*Urochloa panicoides*	Queensland/ Australia	-27,490	151,790	Human observation	[21]
16	*Urochloa panicoides*	Queensland/ Australia	-27,478	151,755	Human observation	[21]
17	*Urochloa panicoides*	Queensland/ Australia	-27,488	151,783	Human observation	[21]
18	*Urochloa panicoides*	Queensland/ Australia	-27,478	151,755	Human observation	[21]
19	*Urochloa panicoides*	Queensland/ Australia	-27,491	151,791	Human observation	[21]
20	*Urochloa panicoides*	Queensland/ Australia	-27,490	151,790	Human observation	[21]
21	*Urochloa panicoides*	Queensland/ Australia	-27,449	151,711	Human observation	[21]
22	*Urochloa panicoides*	Queensland/ Australia	-27,479	151,759	Human observation	[21]
23	*Urochloa panicoides*	Queensland/ Australia	-27,491	151,791	Human observation	[21]
24	*Urochloa panicoides*	Queensland/ Australia	-27,431	151,696	Human observation	[21]
25	*Urochloa panicoides*	Queensland/ Australia	-27,485	151,775	Human observation	[21]
26	*Urochloa panicoides*	Queensland/ Australia	-27,490	151,789	Human observation	[21]
27	*Urochloa panicoides*	Queensland/ Australia	-27,473	151,744	Human observation	[21]
28	*Urochloa panicoides*	Queensland/ Australia	-27,479	151,756	Human observation	[21]
29	*Urochloa panicoides*	New South Wales/ Australia	-29,380	150,090	Human observation	[21]
30	*Urochloa panicoides*	New South Wales/ Australia	-30,268	149,804	Human observation	[21]
31	*Urochloa panicoides*	New South Wales/ Australia	-30,177	149,819	Human observation	[21]
32	*Urochloa panicoides*	New South Wales/ Australia	-29,002	150,334	Human observation	[21]
33	*Urochloa panicoides*	New South Wales/ Australia	-30,234	149,807	Human observation	[21]
34	*Urochloa panicoides*	New South Wales/ Australia	-30,238	149,809	Human observation	[21]
35	*Urochloa panicoides*	New South Wales/ Australia	-29,057	150,314	Human observation	[21]
36	*Urochloa panicoides*	New South Wales/ Australia	-30,238	149,809	Human observation	[21]
37	*Urochloa panicoides*	New South Wales/ Australia	-28,980	150,341	Human observation	[21]
38	*Urochloa panicoides*	New South Wales/ Australia	-32,672	151,299	Human observation	[21]
39	*Urochloa panicoides*	New South Wales/ Australia	-29,409	149,922	Human observation	[21]
40	*Urochloa panicoides*	New South Wales/ Australia	-32,413	149,954	Human observation	[21]

41	*Urochloa panicoides*	New South Wales/ Australia	-30,178	149,819	Human observation	[21]
42	*Urochloa panicoides*	New South Wales/ Australia	-29,398	149,941	Human observation	[21]
43	*Urochloa panicoides*	New South Wales/ Australia	-29,053	150,316	Human observation	[21]
44	*Urochloa panicoides*	New South Wales/ Australia	-29,631	149,822	Human observation	[21]
45	*Urochloa panicoides*	New South Wales/ Australia	-32,304	150,951	Human observation	[21]
46	*Urochloa panicoides*	New South Wales/ Australia	-29,366	150,144	Human observation	[21]
47	*Urochloa panicoides*	New South Wales/ Australia	-33,800	150,982	Human observation	[21]
48	*Urochloa panicoides*	New South Wales/ Australia	-28,979	150,343	Human observation	[21]
49	*Urochloa panicoides*	New South Wales/ Australia	-29,777	149,794	Human observation	[21]
50	*Urochloa panicoides*	New South Wales/ Australia	-32,307	150,942	Human observation	[21]
51	*Urochloa panicoides*	Queensland/ Australia	-27,869	152,029	Human observation	[21]
52	*Urochloa panicoides*	New South Wales/ Australia	-30,566	149,876	Human observation	[21]
53	*Urochloa panicoides*	New South Wales/ Australia	-30,704	150,167	Human observation	[21]
54	*Urochloa panicoides*	New South Wales/ Australia	-30,433	150,321	Human observation	[21]
55	*Urochloa panicoides*	New South Wales/ Australia	-30,818	150,148	Human observation	[21]
56	*Urochloa panicoides*	New South Wales/ Australia	-30,566	149,880	Human observation	[21]
57	*Urochloa panicoides*	New South Wales/ Australia	-31,974	150,921	Human observation	[21]
58	*Urochloa panicoides*	New South Wales/ Australia	-30,533	149,877	Human observation	[21]
59	*Urochloa panicoides*	New South Wales/ Australia	-30,557	149,859	Human observation	[21]
60	*Urochloa panicoides*	New South Wales/ Australia	-30,569	149,871	Human observation	[21]
61	*Urochloa panicoides*	New South Wales/ Australia	-30,570	149,878	Human observation	[21]
62	*Urochloa panicoides*	Gauteng/ South Africa	-25,633	28,212	Human observation	[21]
63	*Urochloa panicoides*	Queensland/ Australia	-27,526	151,850	Human observation	[21]
64	*Urochloa panicoides*	Papua New Guinea	-3,430	142,187	Human observation	[21]
65	*Urochloa panicoides*	Mpumalanga/ South Africa	-25,375	30,985	Human observation	[21]
66	*Urochloa panicoides*	Texas/USA	26,168	-97,742	Human observation	[21]
67	*Urochloa panicoides*	Texas/USA	29,274	-103,305	Human observation	[21]
68	*Urochloa panicoides*	New South Wales/ Australia	-31,409	150,623	Human observation	[21]
69	*Urochloa panicoides*	New South Wales/ Australia	-30,913	150,149	Human observation	[21]
70	*Urochloa panicoides*	New South Wales/ Australia	-31,372	150,654	Human observation	[21]
71	*Urochloa panicoides*	New South Wales/ Australia	-31,380	150,649	Human observation	[21]
72	*Urochloa panicoides*	New South Wales/ Australia	-31,388	150,621	Human observation	[21]
73	*Urochloa panicoides*	New South Wales/ Australia	-31,404	150,620	Human observation	[21]
74	*Urochloa panicoides*	New South Wales/ Australia	-31,408	150,649	Human observation	[21]
75	*Urochloa panicoides*	New South Wales/ Australia	-31,414	150,649	Human observation	[21]
76	*Urochloa panicoides*	New South Wales/ Australia	-31,418	150,619	Human observation	[21]
77	*Urochloa panicoides*	New South Wales/ Australia	-33,415	150,796	Human observation	[21]
78	*Urochloa panicoides*	New South Wales/ Australia	-31,386	150,649	Human observation	[21]
79	*Urochloa panicoides*	New South Wales/ Australia	-31,399	150,616	Human observation	[21]
80	*Urochloa panicoides*	New South Wales/ Australia	-31,395	150,623	Human observation	[21]
81	*Urochloa panicoides*	New South Wales/ Australia	-32,307	149,765	Human observation	[21]
82	*Urochloa panicoides*	New South Wales/ Australia	-30,634	150,043	Human observation	[21]
83	*Urochloa panicoides*	New South Wales/ Australia	-32,274	150,846	Human observation	[21]

84	*Urochloa panicoides*	United Kindom	51,714	-0,337	Human observation	[21]
85	*Urochloa panicoides*	United Kindom	51,732	-0,336	Human observation	[21]
86	*Urochloa panicoides*	Free State/ South Africa	-27,675	25,685	Human observation	[21]
87	*Urochloa panicoides*	New South Wales/ Australia	-30,512	150,125	Human observation	[21]
88	*Urochloa panicoides*	New South Wales/ Australia	-30,544	150,200	Human observation	[21]
89	*Urochloa panicoides*	New South Wales/ Australia	-30,593	150,053	Human observation	[21]
90	*Urochloa panicoides*	New South Wales/ Australia	-30,704	150,361	Human observation	[21]
91	*Urochloa panicoides*	New South Wales/ Australia	-30,564	150,111	Human observation	[21]
92	*Urochloa panicoides*	New South Wales/ Australia	-30,590	150,072	Human observation	[21]
93	*Urochloa panicoides*	New South Wales/ Australia	-30,579	150,072	Human observation	[21]
94	*Urochloa panicoides*	New South Wales/ Australia	-30,589	150,072	Human observation	[21]
95	*Urochloa panicoides*	New South Wales/ Australia	-30,523	150,183	Human observation	[21]
96	*Urochloa panicoides*	New South Wales/ Australia	-30,594	150,085	Human observation	[21]
97	*Urochloa panicoides*	New South Wales/ Australia	-30,589	150,072	Human observation	[21]
98	*Urochloa panicoides*	New South Wales/ Australia	-30,610	150,093	Human observation	[21]
99	*Urochloa panicoides*	New South Wales/ Australia	-30,530	149,875	Human observation	[21]
100	*Urochloa panicoides*	New South Wales/ Australia	-30,521	150,179	Human observation	[21]
101	*Urochloa panicoides*	New South Wales/ Australia	-30,594	150,086	Human observation	[21]
102	*Urochloa panicoides*	New South Wales/ Australia	-30,521	150,178	Human observation	[21]
103	*Urochloa panicoides*	New South Wales/ Australia	-29,140	150,596	Human observation	[21]
104	*Urochloa panicoides*	New South Wales/ Australia	-30,578	150,072	Human observation	[21]
105	*Urochloa panicoides*	New South Wales/ Australia	-30,547	150,190	Human observation	[21]
106	*Urochloa panicoides*	New South Wales/ Australia	-30,386	150,494	Human observation	[21]
107	*Urochloa panicoides*	New South Wales/ Australia	-30,507	150,135	Human observation	[21]
108	*Urochloa panicoides*	New South Wales/ Australia	-30,518	150,126	Human observation	[21]
109	*Urochloa panicoides*	New South Wales/ Australia	-30,547	150,189	Human observation	[21]
110	*Urochloa panicoides*	New South Wales/ Australia	-30,574	150,079	Human observation	[21]
111	*Urochloa panicoides*	New South Wales/ Australia	-30,523	150,183	Human observation	[21]
112	*Urochloa panicoides*	New South Wales/ Australia	-30,605	150,092	Human observation	[21]
113	*Urochloa panicoides*	New South Wales/ Australia	-30,612	150,066	Human observation	[21]
114	*Urochloa panicoides*	New South Wales/ Australia	-31,083	150,099	Human observation	[21]
115	*Urochloa panicoides*	New South Wales/ Australia	-29,527	150,557	Human observation	[21]
116	*Urochloa panicoides*	New South Wales/ Australia	-30,655	150,036	Human observation	[21]
117	*Urochloa panicoides*	New South Wales/ Australia	-30,715	150,362	Human observation	[21]
118	*Urochloa panicoides*	New South Wales/ Australia	-30,533	150,185	Human observation	[21]
119	*Urochloa panicoides*	New South Wales/ Australia	-30,701	150,353	Human observation	[21]
120	*Urochloa panicoides*	New South Wales/ Australia	-30,564	150,111	Human observation	[21]
121	*Urochloa panicoides*	New South Wales/ Australia	-30,529	150,181	Human observation	[21]
122	*Urochloa panicoides*	New South Wales/ Australia	-30,547	150,189	Human observation	[21]
123	*Urochloa panicoides*	New South Wales/ Australia	-31,120	150,071	Human observation	[21]
124	*Urochloa panicoides*	New South Wales/ Australia	-30,386	150,482	Human observation	[21]
125	*Urochloa panicoides*	New South Wales/ Australia	-30,529	150,181	Human observation	[21]
126	*Urochloa panicoides*	New South Wales/ Australia	-30,609	150,044	Human observation	[21]

127	*Urochloa panicoides*	New South Wales/ Australia	-30,529	150,181	Human observation	[21]
128	*Urochloa panicoides*	New South Wales/ Australia	-30,530	149,873	Human observation	[21]
129	*Urochloa panicoides*	New South Wales/ Australia	-30,605	150,091	Human observation	[21]
130	*Urochloa panicoides*	New South Wales/ Australia	-30,726	150,362	Human observation	[21]
131	*Urochloa panicoides*	New South Wales/ Australia	-30,705	150,360	Human observation	[21]
132	*Urochloa panicoides*	New South Wales/ Australia	-31,074	150,077	Human observation	[21]
133	*Urochloa panicoides*	New South Wales/ Australia	-30,614	150,057	Human observation	[21]
134	*Urochloa panicoides*	New South Wales/ Australia	-30,380	150,490	Human observation	[21]
135	*Urochloa panicoides*	New South Wales/ Australia	-30,533	150,184	Human observation	[21]
136	*Urochloa panicoides*	New South Wales/ Australia	-30,564	150,110	Human observation	[21]
137	*Urochloa panicoides*	New South Wales/ Australia	-30,578	150,072	Human observation	[21]
138	*Urochloa panicoides*	New South Wales/ Australia	-30,533	150,184	Human observation	[21]
139	*Urochloa panicoides*	New South Wales/ Australia	-30,506	150,135	Human observation	[21]
140	*Urochloa panicoides*	New South Wales/ Australia	-30,636	150,025	Human observation	[21]
141	*Urochloa panicoides*	New South Wales/ Australia	-30,610	150,076	Human observation	[21]
142	*Urochloa panicoides*	New South Wales/ Australia	-30,610	150,094	Human observation	[21]
143	*Urochloa panicoides*	New South Wales/ Australia	-30,716	150,356	Human observation	[21]
144	*Urochloa panicoides*	New South Wales/ Australia	-30,594	150,086	Human observation	[21]
145	*Urochloa panicoides*	New South Wales/ Australia	-30,605	150,091	Human observation	[21]
146	*Urochloa panicoides*	New South Wales/ Australia	-32,466	151,067	Human observation	[21]
147	*Urochloa panicoides*	New South Wales/ Australia	-30,375	153,098	Human observation	[21]
148	*Urochloa panicoides*	New South Wales/ Australia	-30,726	150,361	Human observation	[21]
149	*Urochloa panicoides*	New South Wales/ Australia	-30,538	150,117	Human observation	[21]
150	*Urochloa panicoides*	New South Wales/ Australia	-30,521	150,179	Human observation	[21]
151	*Urochloa panicoides*	New South Wales/ Australia	-30,589	150,072	Human observation	[21]
152	*Urochloa panicoides*	New South Wales/ Australia	-30,400	150,264	Human observation	[21]
153	*Urochloa panicoides*	New South Wales/ Australia	-30,609	150,044	Human observation	[21]
154	*Urochloa panicoides*	New South Wales/ Australia	-30,552	150,118	Human observation	[21]
155	*Urochloa panicoides*	New South Wales/ Australia	-30,530	149,871	Human observation	[21]
156	*Urochloa panicoides*	New South Wales/ Australia	-30,538	150,117	Human observation	[21]
157	*Urochloa panicoides*	New South Wales/ Australia	-29,337	150,669	Human observation	[21]
158	*Urochloa panicoides*	New South Wales/ Australia	-30,522	150,183	Human observation	[21]
159	*Urochloa panicoides*	New South Wales/ Australia	-30,656	150,016	Human observation	[21]
160	*Urochloa panicoides*	New South Wales/ Australia	-32,650	151,022	Human observation	[21]
161	*Urochloa panicoides*	New South Wales/ Australia	-28,768	153,349	Human observation	[21]
162	*Urochloa panicoides*	New South Wales/ Australia	-29,217	149,119	Human observation	[21]
163	*Urochloa panicoides*	New South Wales/ Australia	-29,465	149,855	Human observation	[21]
164	*Urochloa panicoides*	New South Wales/ Australia	-28,843	153,284	Human observation	[21]
165	*Urochloa panicoides*	New South Wales/ Australia	-29,440	149,871	Human observation	[21]
166	*Urochloa panicoides*	New South Wales/ Australia	-29,399	149,921	Human observation	[21]
167	*Urochloa panicoides*	New South Wales/ Australia	-29,398	149,939	Human observation	[21]
168	*Urochloa panicoides*	New South Wales/ Australia	-29,423	149,897	Human observation	[21]
169	*Urochloa panicoides*	New South Wales/ Australia	-29,468	149,852	Human observation	[21]

170	*Urochloa panicoides*	New South Wales/ Australia	-32,577	148,950	Human observation	[21]
171	*Urochloa panicoides*	New South Wales/ Australia	-32,575	148,952	Human observation	[21]
172	*Urochloa panicoides*	New South Wales/ Australia	-33,775	150,918	Human observation	[21]
173	*Urochloa panicoides*	New South Wales/ Australia	-33,650	150,676	Human observation	[21]
174	*Urochloa panicoides*	New South Wales/ Australia	-30,739	150,179	Human observation	[21]
175	*Urochloa panicoides*	New South Wales/ Australia	-30,629	150,153	Human observation	[21]
176	*Urochloa panicoides*	New South Wales/ Australia	-30,742	150,175	Human observation	[21]
177	*Urochloa panicoides*	New South Wales/ Australia	-30,747	150,174	Human observation	[21]
178	*Urochloa panicoides*	New South Wales/ Australia	-30,741	150,174	Human observation	[21]
179	*Urochloa panicoides*	New South Wales/ Australia	-30,632	150,156	Human observation	[21]
180	*Urochloa panicoides*	New South Wales/ Australia	-30,646	150,168	Human observation	[21]
181	*Urochloa panicoides*	New South Wales/ Australia	-30,630	150,154	Human observation	[21]
182	*Urochloa panicoides*	New South Wales/ Australia	-30,739	150,175	Human observation	[21]
183	*Urochloa panicoides*	New South Wales/ Australia	-30,741	150,175	Human observation	[21]
184	*Urochloa panicoides*	New South Wales/ Australia	-30,741	150,176	Human observation	[21]
185	*Urochloa panicoides*	New South Wales/ Australia	-30,504	149,883	Human observation	[21]
186	*Urochloa panicoides*	New South Wales/ Australia	-30,739	150,177	Human observation	[21]
187	*Urochloa panicoides*	New South Wales/ Australia	-30,741	150,172	Human observation	[21]
188	*Urochloa panicoides*	New South Wales/ Australia	-30,741	150,178	Human observation	[21]
189	*Urochloa panicoides*	New South Wales/ Australia	-30,738	150,173	Human observation	[21]
190	*Urochloa panicoides*	New South Wales/ Australia	-30,738	150,178	Human observation	[21]
191	*Urochloa panicoides*	New South Wales/ Australia	-32,021	150,868	Human observation	[21]
192	*Urochloa panicoides*	New South Wales/ Australia	-30,743	150,176	Human observation	[21]
193	*Urochloa panicoides*	New South Wales/ Australia	-30,477	150,226	Human observation	[21]
194	*Urochloa panicoides*	New South Wales/ Australia	-30,737	150,177	Human observation	[21]
195	*Urochloa panicoides*	New South Wales/ Australia	-30,744	150,175	Human observation	[21]
196	*Urochloa panicoides*	New South Wales/ Australia	-30,738	150,174	Human observation	[21]
197	*Urochloa panicoides*	New South Wales/ Australia	-30,737	150,178	Human observation	[21]
198	*Urochloa panicoides*	New South Wales/ Australia	-30,481	150,243	Human observation	[21]
199	*Urochloa panicoides*	New South Wales/ Australia	-30,632	150,152	Human observation	[21]
200	*Urochloa panicoides*	New South Wales/ Australia	-30,736	150,175	Human observation	[21]
201	*Urochloa panicoides*	New South Wales/ Australia	-30,476	150,236	Human observation	[21]
202	*Urochloa panicoides*	New South Wales/ Australia	-30,485	150,254	Human observation	[21]
203	*Urochloa panicoides*	New South Wales/ Australia	-32,818	151,273	Human observation	[21]
204	*Urochloa panicoides*	New South Wales/ Australia	-32,439	150,088	Human observation	[21]
205	*Urochloa panicoides*	New South Wales/ Australia	-32,271	150,843	Human observation	[21]
206	*Urochloa panicoides*	New South Wales/ Australia	-34,541	150,784	Human observation	[21]
207	*Urochloa panicoides*	New South Wales/ Australia	-32,467	151,134	Human observation	[21]
208	*Urochloa panicoides*	New South Wales/ Australia	-32,281	150,838	Human observation	[21]
209	*Urochloa panicoides*	New South Wales/ Australia	-28,840	150,455	Human observation	[21]
210	*Urochloa panicoides*	New South Wales/ Australia	-32,237	149,581	Human observation	[21]
211	*Urochloa panicoides*	New South Wales/ Australia	-32,273	150,844	Human observation	[21]
212	*Urochloa panicoides*	New South Wales/ Australia	-35,617	145,475	Human observation	[21]

213	*Urochloa panicoides*	New South Wales/ Australia	-32,269	150,842	Human observation	[21]
214	*Urochloa panicoides*	New South Wales/ Australia	-32,500	150,902	Human observation	[21]
215	*Urochloa panicoides*	New South Wales/ Australia	-32,500	150,904	Human observation	[21]
216	*Urochloa panicoides*	New South Wales/ Australia	-29,586	149,233	Human observation	[21]
217	*Urochloa panicoides*	New South Wales/ Australia	-32,499	150,903	Human observation	[21]
218	*Urochloa panicoides*	New South Wales/ Australia	-31,125	150,929	Human observation	[21]
219	*Urochloa panicoides*	New South Wales/ Australia	-32,500	150,902	Human observation	[21]
220	*Urochloa panicoides*	New South Wales/ Australia	-33,919	151,176	Human observation	[21]
221	*Urochloa panicoides*	New South Wales/ Australia	-29,336	149,301	Human observation	[21]
222	*Urochloa panicoides*	New South Wales/ Australia	-32,272	150,845	Human observation	[21]
223	*Urochloa panicoides*	New South Wales/ Australia	-29,346	149,331	Human observation	[21]
224	*Urochloa panicoides*	New South Wales/ Australia	-32,283	150,838	Human observation	[21]
225	*Urochloa panicoides*	New South Wales/ Australia	-32,609	151,985	Human observation	[21]
226	*Urochloa panicoides*	New South Wales/ Australia	-32,299	150,877	Human observation	[21]
227	*Urochloa panicoides*	New South Wales/ Australia	-32,284	150,835	Human observation	[21]
228	*Urochloa panicoides*	New South Wales/ Australia	-32,286	150,834	Human observation	[21]
229	*Urochloa panicoides*	New South Wales/ Australia	-32,265	150,849	Human observation	[21]
230	*Urochloa panicoides*	New South Wales/ Australia	-33,808	150,710	Human observation	[21]
231	*Urochloa panicoides*	New South Wales/ Australia	-33,801	150,724	Human observation	[21]
232	*Urochloa panicoides*	New South Wales/ Australia	-30,310	149,762	Human observation	[21]
233	*Urochloa panicoides*	New South Wales/ Australia	-32,369	150,744	Human observation	[21]
234	*Urochloa panicoides*	New South Wales/ Australia	-32,271	150,844	Human observation	[21]
235	*Urochloa panicoides*	Kenya	1,205	37,305	Human observation	[21]
236	*Urochloa panicoides*	Kenya	0,261	37,497	Human observation	[21]
237	*Urochloa panicoides*	Kenya	0,259	37,500	Human observation	[21]
238	*Urochloa panicoides*	Kenya	0,231	37,467	Human observation	[21]
239	*Urochloa panicoides*	Kenya	0,262	37,497	Human observation	[21]
240	*Urochloa panicoides*	Kenya	0,260	37,497	Human observation	[21]
241	*Urochloa panicoides*	Kenya	0,249	37,488	Human observation	[21]
242	*Urochloa panicoides*	India	10,500	79,500	Human observation	[21]
243	*Urochloa panicoides*	South Australia	-35,572	138,591	Human observation	[21]
244	*Urochloa panicoides*	New South Wales/ Australia	-32,351	152,314	Human observation	[21]
245	*Urochloa panicoides*	New South Wales/ Australia	-30,605	150,091	Human observation	[21]
246	*Urochloa panicoides*	New South Wales/ Australia	-30,538	150,117	Human observation	[21]
247	*Urochloa panicoides*	New South Wales/ Australia	-30,444	150,324	Human observation	[21]
248	*Urochloa panicoides*	New South Wales/ Australia	-28,737	150,420	Human observation	[21]
249	*Urochloa panicoides*	New South Wales/ Australia	-30,578	150,072	Human observation	[21]
250	*Urochloa panicoides*	New South Wales/ Australia	-32,746	151,528	Human observation	[21]
251	*Urochloa panicoides*	New South Wales/ Australia	-30,529	150,181	Human observation	[21]
252	*Urochloa panicoides*	New South Wales/ Australia	-30,261	149,736	Human observation	[21]
253	*Urochloa panicoides*	New South Wales/ Australia	-30,538	150,117	Human observation	[21]
254	*Urochloa panicoides*	New South Wales/ Australia	-30,547	150,190	Human observation	[21]
255	*Urochloa panicoides*	New South Wales/ Australia	-30,589	150,072	Human observation	[21]

256	*Urochloa panicoides*	New South Wales/ Australia	-30,609	150,044	Human observation	[21]
257	*Urochloa panicoides*	New South Wales/ Australia	-29,203	151,336	Human observation	[21]
258	*Urochloa panicoides*	New South Wales/ Australia	-28,679	150,439	Human observation	[21]
259	*Urochloa panicoides*	New South Wales/ Australia	-29,204	151,334	Human observation	[21]
260	*Urochloa panicoides*	New South Wales/ Australia	-29,463	149,945	Human observation	[21]
261	*Urochloa panicoides*	New South Wales/ Australia	-30,699	150,053	Human observation	[21]
262	*Urochloa panicoides*	New South Wales/ Australia	-30,450	150,325	Human observation	[21]
263	*Urochloa panicoides*	New South Wales/ Australia	-33,875	150,762	Human observation	[21]
264	*Urochloa panicoides*	New South Wales/ Australia	-30,589	150,072	Human observation	[21]
265	*Urochloa panicoides*	New South Wales/ Australia	-30,579	150,072	Human observation	[21]
266	*Urochloa panicoides*	New South Wales/ Australia	-31,765	152,671	Human observation	[21]
267	*Urochloa panicoides*	New South Wales/ Australia	-30,589	150,072	Human observation	[21]
268	*Urochloa panicoides*	New South Wales/ Australia	-28,681	150,433	Human observation	[21]
269	*Urochloa panicoides*	New South Wales/ Australia	-30,271	149,773	Human observation	[21]
270	*Urochloa panicoides*	New South Wales/ Australia	-29,429	149,913	Human observation	[21]
271	*Urochloa panicoides*	New South Wales/ Australia	-29,428	149,905	Human observation	[21]
272	*Urochloa panicoides*	New South Wales/ Australia	-30,610	150,094	Human observation	[21]
273	*Urochloa panicoides*	New South Wales/ Australia	-33,438	151,373	Human observation	[21]
274	*Urochloa panicoides*	New South Wales/ Australia	-30,636	150,025	Human observation	[21]
275	*Urochloa panicoides*	New South Wales/ Australia	-30,538	150,117	Human observation	[21]
276	*Urochloa panicoides*	New South Wales/ Australia	-28,678	150,437	Human observation	[21]
277	*Urochloa panicoides*	New South Wales/ Australia	-30,547	150,189	Human observation	[21]
278	*Urochloa panicoides*	New South Wales/ Australia	-30,578	150,072	Human observation	[21]
279	*Urochloa panicoides*	New South Wales/ Australia	-29,435	149,895	Human observation	[21]
280	*Urochloa panicoides*	New South Wales/ Australia	-29,346	149,330	Human observation	[21]
281	*Urochloa panicoides*	New South Wales/ Australia	-30,564	150,111	Human observation	[21]
282	*Urochloa panicoides*	Eastern Cape/ South Africa	-32,591	25,145	Human observation	[21]
283	*Urochloa panicoides*	Sudan	15,033	29,150	Human observation	[21]
284	*Urochloa panicoides*	New South Wales/ Australia	-30,449	148,694	Human observation	[21]
285	*Urochloa panicoides*	New South Wales/ Australia	-31,746	150,357	Human observation	[21]
286	*Urochloa panicoides*	New South Wales/ Australia	-30,910	150,645	Human observation	[21]
287	*Urochloa panicoides*	New South Wales/ Australia	-30,156	148,937	Human observation	[21]
288	*Urochloa panicoides*	New South Wales/ Australia	-30,253	149,638	Human observation	[21]
289	*Urochloa panicoides*	New South Wales/ Australia	-29,330	150,861	Human observation	[21]
290	*Urochloa panicoides*	New South Wales/ Australia	-30,141	148,961	Human observation	[21]
291	*Urochloa panicoides*	New South Wales/ Australia	-29,792	150,701	Human observation	[21]
292	*Urochloa panicoides*	New South Wales/ Australia	-30,076	148,960	Human observation	[21]
293	*Urochloa panicoides*	New South Wales/ Australia	-30,976	150,452	Human observation	[21]
294	*Urochloa panicoides*	New South Wales/ Australia	-31,989	149,387	Human observation	[21]
295	*Urochloa panicoides*	New South Wales/ Australia	-32,399	148,818	Human observation	[21]
296	*Urochloa panicoides*	New South Wales/ Australia	-30,156	148,937	Human observation	[21]
297	*Urochloa panicoides*	New South Wales/ Australia	-33,838	151,018	Human observation	[21]
298	*Urochloa panicoides*	New South Wales/ Australia	-30,076	148,960	Human observation	[21]

299	*Urochloa panicoides*	New South Wales/ Australia	-33,875	150,731	Human observation	[21]
300	*Urochloa panicoides*	New South Wales/ Australia	-29,211	151,310	Human observation	[21]
301	*Urochloa panicoides*	New South Wales/ Australia	-30,449	148,694	Human observation	[21]
302	*Urochloa panicoides*	New South Wales/ Australia	-33,150	150,117	Human observation	[21]
303	*Urochloa panicoides*	New South Wales/ Australia	-30,141	148,961	Human observation	[21]
304	*Urochloa panicoides*	New South Wales/ Australia	-30,236	149,448	Human observation	[21]
305	*Urochloa panicoides*	New South Wales/ Australia	-31,989	149,391	Human observation	[21]
306	*Urochloa panicoides*	New South Wales/ Australia	-30,692	150,049	Human observation	[21]
307	*Urochloa panicoides*	New South Wales/ Australia	-32,359	151,086	Human observation	[21]
308	*Urochloa panicoides*	New South Wales/ Australia	-32,400	148,819	Human observation	[21]
309	*Urochloa panicoides*	New South Wales/ Australia	-31,099	149,915	Human observation	[21]
310	*Urochloa panicoides*	New South Wales/ Australia	-30,966	150,237	Human observation	[21]
311	*Urochloa panicoides*	New South Wales/ Australia	-32,628	151,077	Human observation	[21]
312	*Urochloa panicoides*	New South Wales/ Australia	-32,540	151,004	Human observation	[21]
313	*Urochloa panicoides*	New South Wales/ Australia	-32,530	151,036	Human observation	[21]
314	*Urochloa panicoides*	New South Wales/ Australia	-30,278	149,785	Human observation	[21]
315	*Urochloa panicoides*	New South Wales/ Australia	-31,767	151,068	Human observation	[21]
316	*Urochloa panicoides*	New South Wales/ Australia	-32,445	150,850	Human observation	[21]
317	*Urochloa panicoides*	Queensland/ Australia	-23,765	150,351	Human observation	[21]
318	*Urochloa panicoides*	Jalisco/ Mexico	19,747	-104,165	Human observation	[21]
319	*Urochloa panicoides*	New South Wales/ Australia	-31,894	152,472	Human observation	[21]
320	*Urochloa panicoides*	Belgium	50,939	3,138	Human observation	[21]
321	*Urochloa panicoides*	New South Wales/ Australia	-32,468	150,655	Human observation	[21]
322	*Urochloa panicoides*	New South Wales/ Australia	-30,883	150,673	Human observation	[21]
323	*Urochloa panicoides*	New South Wales/ Australia	-30,749	150,184	Human observation	[21]
324	*Urochloa panicoides*	New South Wales/ Australia	-30,630	150,154	Human observation	[21]
325	*Urochloa panicoides*	New South Wales/ Australia	-30,512	149,903	Human observation	[21]
326	*Urochloa panicoides*	New South Wales/ Australia	-32,265	150,847	Human observation	[21]
327	*Urochloa panicoides*	New South Wales/ Australia	-32,265	150,849	Human observation	[21]
328	*Urochloa panicoides*	New South Wales/ Australia	-32,274	150,840	Human observation	[21]
329	*Urochloa panicoides*	New South Wales/ Australia	-32,050	150,858	Human observation	[21]
330	*Urochloa panicoides*	New South Wales/ Australia	-31,892	152,472	Human observation	[21]
331	*Urochloa panicoides*	New South Wales/ Australia	-33,739	151,140	Human observation	[21]
332	*Urochloa panicoides*	New South Wales/ Australia	-32,320	150,651	Human observation	[21]
333	*Urochloa panicoides*	New South Wales/ Australia	-33,807	150,671	Human observation	[21]
334	*Urochloa panicoides*	New South Wales/ Australia	-33,809	150,673	Human observation	[21]
335	*Urochloa panicoides*	New South Wales/ Australia	-29,577	152,551	Human observation	[21]
336	*Urochloa panicoides*	New South Wales/ Australia	-32,053	150,860	Human observation	[21]
337	*Urochloa panicoides*	New South Wales/ Australia	-33,768	150,909	Human observation	[21]
338	*Urochloa panicoides*	New South Wales/ Australia	-32,214	148,828	Human observation	[21]
339	*Urochloa panicoides*	New South Wales/ Australia	-32,310	150,824	Human observation	[21]
340	*Urochloa panicoides*	New South Wales/ Australia	-32,304	150,836	Human observation	[21]
341	*Urochloa panicoides*	New South Wales/ Australia	-30,972	150,342	Human observation	[21]

342	*Urochloa panicoides*	New South Wales/ Australia	-30,537	150,023	Human observation	[21]
343	*Urochloa panicoides*	New South Wales/ Australia	-32,700	151,071	Human observation	[21]
344	*Urochloa panicoides*	New South Wales/ Australia	-32,408	151,043	Human observation	[21]
345	*Urochloa panicoides*	New South Wales/ Australia	-32,452	151,065	Human observation	[21]
346	*Urochloa panicoides*	New South Wales/ Australia	-32,284	150,733	Human observation	[21]
347	*Urochloa panicoides*	New South Wales/ Australia	-32,297	150,734	Human observation	[21]
348	*Urochloa panicoides*	New South Wales/ Australia	-32,321	150,650	Human observation	[21]
349	*Urochloa panicoides*	New South Wales/ Australia	-31,767	150,835	Human observation	[21]
350	*Urochloa panicoides*	New South Wales/ Australia	-31,921	150,637	Human observation	[21]
351	*Urochloa panicoides*	New South Wales/ Australia	-32,193	150,097	Human observation	[21]
352	*Urochloa panicoides*	New South Wales/ Australia	-32,015	150,567	Human observation	[21]
353	*Urochloa panicoides*	New South Wales/ Australia	-32,018	150,567	Human observation	[21]
354	*Urochloa panicoides*	New South Wales/ Australia	-32,312	150,914	Human observation	[21]
355	*Urochloa panicoides*	New South Wales/ Australia	-29,626	150,576	Human observation	[21]
356	*Urochloa panicoides*	New South Wales/ Australia	-29,727	150,606	Human observation	[21]
357	*Urochloa panicoides*	New South Wales/ Australia	-31,965	149,904	Human observation	[21]
358	*Urochloa panicoides*	New South Wales/ Australia	-31,967	149,892	Human observation	[21]
359	*Urochloa panicoides*	New South Wales/ Australia	-31,854	149,350	Human observation	[21]
360	*Urochloa panicoides*	New South Wales/ Australia	-31,882	149,784	Human observation	[21]
361	*Urochloa panicoides*	New South Wales/ Australia	-32,253	148,856	Human observation	[21]
362	*Urochloa panicoides*	New South Wales/ Australia	-30,015	151,942	Human observation	[21]
363	*Urochloa panicoides*	New South Wales/ Australia	-29,776	148,397	Human observation	[21]
364	*Urochloa panicoides*	New South Wales/ Australia	-29,737	148,401	Human observation	[21]
365	*Urochloa panicoides*	New South Wales/ Australia	-29,740	148,408	Human observation	[21]
366	*Urochloa panicoides*	New South Wales/ Australia	-28,766	150,766	Human observation	[21]
367	*Urochloa panicoides*	New South Wales/ Australia	-28,788	150,813	Human observation	[21]
368	*Urochloa panicoides*	New South Wales/ Australia	-32,090	150,115	Human observation	[21]
369	*Urochloa panicoides*	New South Wales/ Australia	-32,086	150,128	Human observation	[21]
370	*Urochloa panicoides*	New South Wales/ Australia	-32,091	150,130	Human observation	[21]
371	*Urochloa panicoides*	New South Wales/ Australia	-32,086	150,120	Human observation	[21]
372	*Urochloa panicoides*	New South Wales/ Australia	-32,087	150,118	Human observation	[21]
373	*Urochloa panicoides*	New South Wales/ Australia	-32,094	150,121	Human observation	[21]
374	*Urochloa panicoides*	New South Wales/ Australia	-32,089	150,116	Human observation	[21]
375	*Urochloa panicoides*	New South Wales/ Australia	-32,091	150,125	Human observation	[21]
376	*Urochloa panicoides*	New South Wales/ Australia	-32,079	150,140	Human observation	[21]
377	*Urochloa panicoides*	New South Wales/ Australia	-32,083	150,138	Human observation	[21]
378	*Urochloa panicoides*	New South Wales/ Australia	-32,091	150,141	Human observation	[21]
379	*Urochloa panicoides*	New South Wales/ Australia	-32,090	150,141	Human observation	[21]
380	*Urochloa panicoides*	New South Wales/ Australia	-32,106	150,127	Human observation	[21]
381	*Urochloa panicoides*	New South Wales/ Australia	-32,104	150,122	Human observation	[21]
382	*Urochloa panicoides*	New South Wales/ Australia	-32,080	150,136	Human observation	[21]
383	*Urochloa panicoides*	New South Wales/ Australia	-30,040	150,602	Human observation	[21]
384	*Urochloa panicoides*	New South Wales/ Australia	-29,741	150,977	Human observation	[21]

385	*Urochloa panicoides*	New South Wales/ Australia	-29,385	150,126	Human observation	[21]
386	*Urochloa panicoides*	New South Wales/ Australia	-30,077	150,619	Human observation	[21]
387	*Urochloa panicoides*	New South Wales/ Australia	-30,077	150,619	Human observation	[21]
388	*Urochloa panicoides*	New South Wales/ Australia	-30,025	150,602	Human observation	[21]
389	*Urochloa panicoides*	New South Wales/ Australia	-30,025	150,602	Human observation	[21]
390	*Urochloa panicoides*	New South Wales/ Australia	-29,698	150,688	Human observation	[21]
391	*Urochloa panicoides*	New South Wales/ Australia	-29,745	150,882	Human observation	[21]
392	*Urochloa panicoides*	New South Wales/ Australia	-29,777	150,642	Human observation	[21]
393	*Urochloa panicoides*	New South Wales/ Australia	-30,205	149,576	Human observation	[21]
394	*Urochloa panicoides*	New South Wales/ Australia	-30,169	149,744	Human observation	[21]
395	*Urochloa panicoides*	New South Wales/ Australia	-31,278	149,777	Human observation	[21]
396	*Urochloa panicoides*	New South Wales/ Australia	-30,145	149,701	Human observation	[21]
397	*Urochloa panicoides*	New South Wales/ Australia	-30,977	150,367	Human observation	[21]
398	*Urochloa panicoides*	New South Wales/ Australia	-30,930	150,627	Human observation	[21]
399	*Urochloa panicoides*	New South Wales/ Australia	-30,696	150,502	Human observation	[21]
400	*Urochloa panicoides*	New South Wales/ Australia	-31,665	150,403	Human observation	[21]
401	*Urochloa panicoides*	New South Wales/ Australia	-31,646	150,718	Human observation	[21]
402	*Urochloa panicoides*	New South Wales/ Australia	-31,441	150,591	Human observation	[21]
403	*Urochloa panicoides*	New South Wales/ Australia	-32,067	150,818	Human observation	[21]
404	*Urochloa panicoides*	New South Wales/ Australia	-30,080	150,620	Human observation	[21]
405	*Urochloa panicoides*	New South Wales/ Australia	-30,076	150,617	Human observation	[21]
406	*Urochloa panicoides*	New South Wales/ Australia	-30,076	150,617	Human observation	[21]
407	*Urochloa panicoides*	New South Wales/ Australia	-29,741	150,977	Human observation	[21]
408	*Urochloa panicoides*	New South Wales/ Australia	-29,710	150,680	Human observation	[21]
409	*Urochloa panicoides*	New South Wales/ Australia	-29,703	150,665	Human observation	[21]
410	*Urochloa panicoides*	New South Wales/ Australia	-32,181	150,309	Human observation	[21]
411	*Urochloa panicoides*	New South Wales/ Australia	-32,181	150,309	Human observation	[21]
412	*Urochloa panicoides*	New South Wales/ Australia	-32,467	150,656	Human observation	[21]
413	*Urochloa panicoides*	New South Wales/ Australia	-30,477	150,711	Human observation	[21]
414	*Urochloa panicoides*	New South Wales/ Australia	-30,018	150,731	Human observation	[21]
415	*Urochloa panicoides*	New South Wales/ Australia	-29,871	151,017	Human observation	[21]
416	*Urochloa panicoides*	New South Wales/ Australia	-29,072	151,250	Human observation	[21]
417	*Urochloa panicoides*	New South Wales/ Australia	-29,072	151,249	Human observation	[21]
418	*Urochloa panicoides*	New South Wales/ Australia	-29,790	150,694	Human observation	[21]
419	*Urochloa panicoides*	New South Wales/ Australia	-29,786	150,535	Human observation	[21]
420	*Urochloa panicoides*	New South Wales/ Australia	-30,019	150,623	Human observation	[21]
421	*Urochloa panicoides*	New South Wales/ Australia	-29,547	150,535	Human observation	[21]
422	*Urochloa panicoides*	New South Wales/ Australia	-29,727	150,539	Human observation	[21]
423	*Urochloa panicoides*	New South Wales/ Australia	-29,543	151,132	Human observation	[21]
424	*Urochloa panicoides*	New South Wales/ Australia	-29,337	150,669	Human observation	[21]
425	*Urochloa panicoides*	New South Wales/ Australia	-29,449	150,922	Human observation	[21]
426	*Urochloa panicoides*	New South Wales/ Australia	-30,083	149,949	Human observation	[21]
427	*Urochloa panicoides*	New South Wales/ Australia	-31,988	150,177	Human observation	[21]

428	*Urochloa panicoides*	New South Wales/ Australia	-31,740	150,266	Human observation	[21]
429	*Urochloa panicoides*	New South Wales/ Australia	-29,033	150,204	Human observation	[21]
430	*Urochloa panicoides*	New South Wales/ Australia	-29,154	150,359	Human observation	[21]
431	*Urochloa panicoides*	New South Wales/ Australia	-29,642	150,015	Human observation	[21]
432	*Urochloa panicoides*	New South Wales/ Australia	-29,523	150,191	Human observation	[21]
433	*Urochloa panicoides*	New South Wales/ Australia	-29,658	150,036	Human observation	[21]
434	*Urochloa panicoides*	New South Wales/ Australia	-29,515	150,085	Human observation	[21]
435	*Urochloa panicoides*	New South Wales/ Australia	-29,919	150,245	Human observation	[21]
436	*Urochloa panicoides*	New South Wales/ Australia	-29,939	150,065	Human observation	[21]
437	*Urochloa panicoides*	New South Wales/ Australia	-29,771	150,111	Human observation	[21]
438	*Urochloa panicoides*	New South Wales/ Australia	-29,768	150,111	Human observation	[21]
439	*Urochloa panicoides*	New South Wales/ Australia	-29,847	150,148	Human observation	[21]
440	*Urochloa panicoides*	New South Wales/ Australia	-29,847	150,149	Human observation	[21]
441	*Urochloa panicoides*	New South Wales/ Australia	-29,985	150,210	Human observation	[21]
442	*Urochloa panicoides*	New South Wales/ Australia	-29,980	150,212	Human observation	[21]
443	*Urochloa panicoides*	New South Wales/ Australia	-29,762	150,168	Human observation	[21]
444	*Urochloa panicoides*	New South Wales/ Australia	-29,762	150,118	Human observation	[21]
445	*Urochloa panicoides*	New South Wales/ Australia	-29,737	150,165	Human observation	[21]
446	*Urochloa panicoides*	New South Wales/ Australia	-29,608	150,224	Human observation	[21]
447	*Urochloa panicoides*	New South Wales/ Australia	-29,537	150,213	Human observation	[21]
448	*Urochloa panicoides*	New South Wales/ Australia	-29,701	150,080	Human observation	[21]
449	*Urochloa panicoides*	New South Wales/ Australia	-29,509	150,167	Human observation	[21]
450	*Urochloa panicoides*	New South Wales/ Australia	-29,660	150,094	Human observation	[21]
451	*Urochloa panicoides*	New South Wales/ Australia	-29,665	150,041	Human observation	[21]
452	*Urochloa panicoides*	New South Wales/ Australia	-29,664	150,036	Human observation	[21]
453	*Urochloa panicoides*	New South Wales/ Australia	-29,687	150,455	Human observation	[21]
454	*Urochloa panicoides*	New South Wales/ Australia	-29,514	150,062	Human observation	[21]
455	*Urochloa panicoides*	New South Wales/ Australia	-29,636	150,381	Human observation	[21]
456	*Urochloa panicoides*	New South Wales/ Australia	-29,611	150,371	Human observation	[21]
457	*Urochloa panicoides*	New South Wales/ Australia	-29,644	150,303	Human observation	[21]
458	*Urochloa panicoides*	New South Wales/ Australia	-29,589	150,348	Human observation	[21]
459	*Urochloa panicoides*	New South Wales/ Australia	-29,522	149,950	Human observation	[21]
460	*Urochloa panicoides*	New South Wales/ Australia	-32,244	150,045	Human observation	[21]
461	*Urochloa panicoides*	New South Wales/ Australia	-32,194	150,095	Human observation	[21]
462	*Urochloa panicoides*	New South Wales/ Australia	-29,248	150,079	Human observation	[21]
463	*Urochloa panicoides*	New South Wales/ Australia	-29,248	150,078	Human observation	[21]
464	*Urochloa panicoides*	New South Wales/ Australia	-29,504	151,116	Human observation	[21]
465	*Urochloa panicoides*	New South Wales/ Australia	-29,507	151,115	Human observation	[21]
466	*Urochloa panicoides*	New South Wales/ Australia	-29,499	151,078	Human observation	[21]
467	*Urochloa panicoides*	New South Wales/ Australia	-29,822	150,739	Human observation	[21]
468	*Urochloa panicoides*	New South Wales/ Australia	-29,858	150,535	Human observation	[21]
469	*Urochloa panicoides*	New South Wales/ Australia	-29,526	149,858	Human observation	[21]
470	*Urochloa panicoides*	New South Wales/ Australia	-29,775	150,019	Human observation	[21]

471	*Urochloa panicoides*	New South Wales/ Australia	-29,522	149,669	Human observation	[21]
472	*Urochloa panicoides*	New South Wales/ Australia	-30,114	150,784	Human observation	[21]
473	*Urochloa panicoides*	New South Wales/ Australia	-30,113	150,785	Human observation	[21]
474	*Urochloa panicoides*	New South Wales/ Australia	-29,649	150,011	Human observation	[21]
475	*Urochloa panicoides*	New South Wales/ Australia	-29,672	150,642	Human observation	[21]
476	*Urochloa panicoides*	New South Wales/ Australia	-29,888	150,759	Human observation	[21]
477	*Urochloa panicoides*	New South Wales/ Australia	-29,875	150,746	Human observation	[21]
478	*Urochloa panicoides*	New South Wales/ Australia	-29,876	150,745	Human observation	[21]
479	*Urochloa panicoides*	New South Wales/ Australia	-29,557	150,429	Human observation	[21]
480	*Urochloa panicoides*	New South Wales/ Australia	-29,555	150,428	Human observation	[21]
481	*Urochloa panicoides*	New South Wales/ Australia	-32,763	151,371	Human observation	[21]
482	*Urochloa panicoides*	New South Wales/ Australia	-32,762	151,371	Human observation	[21]
483	*Urochloa panicoides*	New South Wales/ Australia	-28,978	151,275	Human observation	[21]
484	*Urochloa panicoides*	New South Wales/ Australia	-31,278	149,373	Human observation	[21]
485	*Urochloa panicoides*	New South Wales/ Australia	-29,642	150,015	Human observation	[21]
486	*Urochloa panicoides*	New South Wales/ Australia	-31,019	149,976	Human observation	[21]
487	*Urochloa panicoides*	New South Wales/ Australia	-31,928	149,920	Human observation	[21]
488	*Urochloa panicoides*	New South Wales/ Australia	-29,491	150,078	Human observation	[21]
489	*Urochloa panicoides*	New South Wales/ Australia	-30,278	149,818	Human observation	[21]
490	*Urochloa panicoides*	New South Wales/ Australia	-30,051	149,667	Human observation	[21]
491	*Urochloa panicoides*	New South Wales/ Australia	-31,448	150,616	Human observation	[21]
492	*Urochloa panicoides*	New South Wales/ Australia	-31,182	150,757	Human observation	[21]
493	*Urochloa panicoides*	New South Wales/ Australia	-31,378	150,535	Human observation	[21]
494	*Urochloa panicoides*	New South Wales/ Australia	-31,454	150,586	Human observation	[21]
495	*Urochloa panicoides*	New South Wales/ Australia	-31,344	150,514	Human observation	[21]
496	*Urochloa panicoides*	New South Wales/ Australia	-30,984	150,762	Human observation	[21]
497	*Urochloa panicoides*	New South Wales/ Australia	-30,985	150,771	Human observation	[21]
498	*Urochloa panicoides*	New South Wales/ Australia	-30,932	150,531	Human observation	[21]
499	*Urochloa panicoides*	New South Wales/ Australia	-30,966	150,770	Human observation	[21]
500	*Urochloa panicoides*	New South Wales/ Australia	-30,028	150,591	Human observation	[21]
501	*Urochloa panicoides*	New South Wales/ Australia	-31,182	149,892	Human observation	[21]
502	*Urochloa panicoides*	New South Wales/ Australia	-31,046	149,742	Human observation	[21]
503	*Urochloa panicoides*	New South Wales/ Australia	-31,110	149,954	Human observation	[21]
504	*Urochloa panicoides*	New South Wales/ Australia	-32,069	149,965	Human observation	[21]
505	*Urochloa panicoides*	New South Wales/ Australia	-32,087	149,563	Human observation	[21]
506	*Urochloa panicoides*	New South Wales/ Australia	-31,748	149,224	Human observation	[21]
507	*Urochloa panicoides*	New South Wales/ Australia	-31,810	149,463	Human observation	[21]
508	*Urochloa panicoides*	New South Wales/ Australia	-31,275	149,394	Human observation	[21]
509	*Urochloa panicoides*	New South Wales/ Australia	-31,262	149,379	Human observation	[21]
510	*Urochloa panicoides*	New South Wales/ Australia	-31,261	149,376	Human observation	[21]
511	*Urochloa panicoides*	New South Wales/ Australia	-31,487	149,947	Human observation	[21]
512	*Urochloa panicoides*	New South Wales/ Australia	-31,210	149,637	Human observation	[21]
513	*Urochloa panicoides*	New South Wales/ Australia	-31,081	149,807	Human observation	[21]

514	*Urochloa panicoides*	New South Wales/ Australia	-31,032	149,955	Human observation	[21]
515	*Urochloa panicoides*	New South Wales/ Australia	-29,790	150,700	Human observation	[21]
516	*Urochloa panicoides*	New South Wales/ Australia	-29,832	150,694	Human observation	[21]
517	*Urochloa panicoides*	New South Wales/ Australia	-29,383	149,819	Human observation	[21]
518	*Urochloa panicoides*	New South Wales/ Australia	-32,070	150,594	Human observation	[21]
519	*Urochloa panicoides*	New South Wales/ Australia	-30,083	149,949	Human observation	[21]
520	*Urochloa panicoides*	New South Wales/ Australia	-29,738	150,137	Human observation	[21]
521	*Urochloa panicoides*	New South Wales/ Australia	-29,406	150,135	Human observation	[21]
522	*Urochloa panicoides*	New South Wales/ Australia	-29,864	150,208	Human observation	[21]
523	*Urochloa panicoides*	New South Wales/ Australia	-29,905	150,076	Human observation	[21]
524	*Urochloa panicoides*	New South Wales/ Australia	-29,127	149,227	Human observation	[21]
525	*Urochloa panicoides*	New South Wales/ Australia	-29,311	149,087	Human observation	[21]
526	*Urochloa panicoides*	New South Wales/ Australia	-29,277	149,785	Human observation	[21]
527	*Urochloa panicoides*	New South Wales/ Australia	-29,275	149,287	Human observation	[21]
528	*Urochloa panicoides*	New South Wales/ Australia	-29,467	149,533	Human observation	[21]
529	*Urochloa panicoides*	New South Wales/ Australia	-29,471	149,914	Human observation	[21]
530	*Urochloa panicoides*	New South Wales/ Australia	-29,655	149,234	Human observation	[21]
531	*Urochloa panicoides*	New South Wales/ Australia	-29,520	149,948	Human observation	[21]
532	*Urochloa panicoides*	New South Wales/ Australia	-29,632	149,533	Human observation	[21]
533	*Urochloa panicoides*	New South Wales/ Australia	-29,544	149,635	Human observation	[21]
534	*Urochloa panicoides*	New South Wales/ Australia	-29,728	149,799	Human observation	[21]
535	*Urochloa panicoides*	New South Wales/ Australia	-29,729	149,450	Human observation	[21]
536	*Urochloa panicoides*	New South Wales/ Australia	-29,860	149,901	Human observation	[21]
537	*Urochloa panicoides*	New South Wales/ Australia	-28,896	149,691	Human observation	[21]
538	*Urochloa panicoides*	New South Wales/ Australia	-28,710	150,278	Human observation	[21]
539	*Urochloa panicoides*	New South Wales/ Australia	-28,843	150,237	Human observation	[21]
540	*Urochloa panicoides*	New South Wales/ Australia	-29,107	148,953	Human observation	[21]
541	*Urochloa panicoides*	New South Wales/ Australia	-29,163	148,848	Human observation	[21]
542	*Urochloa panicoides*	New South Wales/ Australia	-29,357	148,825	Human observation	[21]
543	*Urochloa panicoides*	New South Wales/ Australia	-29,291	148,817	Human observation	[21]
544	*Urochloa panicoides*	New South Wales/ Australia	-29,373	148,854	Human observation	[21]
545	*Urochloa panicoides*	New South Wales/ Australia	-29,458	149,183	Human observation	[21]
546	*Urochloa panicoides*	New South Wales/ Australia	-29,422	148,754	Human observation	[21]
547	*Urochloa panicoides*	New South Wales/ Australia	-29,587	148,694	Human observation	[21]
548	*Urochloa panicoides*	New South Wales/ Australia	-29,486	148,851	Human observation	[21]
549	*Urochloa panicoides*	New South Wales/ Australia	-29,721	148,897	Human observation	[21]
550	*Urochloa panicoides*	New South Wales/ Australia	-29,810	148,909	Human observation	[21]
551	*Urochloa panicoides*	New South Wales/ Australia	-29,776	148,843	Human observation	[21]
552	*Urochloa panicoides*	New South Wales/ Australia	-30,093	148,956	Human observation	[21]
553	*Urochloa panicoides*	New South Wales/ Australia	-30,202	148,820	Human observation	[21]
554	*Urochloa panicoides*	New South Wales/ Australia	-30,768	150,432	Human observation	[21]
555	*Urochloa panicoides*	New South Wales/ Australia	-30,505	150,091	Human observation	[21]
556	*Urochloa panicoides*	New South Wales/ Australia	-30,668	150,441	Human observation	[21]

557	*Urochloa panicoides*	New South Wales/ Australia	-30,716	150,469	Human observation	[21]
558	*Urochloa panicoides*	New South Wales/ Australia	-30,828	150,341	Human observation	[21]
559	*Urochloa panicoides*	New South Wales/ Australia	-30,779	150,071	Human observation	[21]
560	*Urochloa panicoides*	New South Wales/ Australia	-30,908	150,051	Human observation	[21]
561	*Urochloa panicoides*	New South Wales/ Australia	-30,958	150,421	Human observation	[21]
562	*Urochloa panicoides*	New South Wales/ Australia	-30,777	150,568	Human observation	[21]
563	*Urochloa panicoides*	New South Wales/ Australia	-30,767	150,821	Human observation	[21]
564	*Urochloa panicoides*	New South Wales/ Australia	-30,770	150,611	Human observation	[21]
565	*Urochloa panicoides*	New South Wales/ Australia	-29,066	151,503	Human observation	[21]
566	*Urochloa panicoides*	New South Wales/ Australia	-29,169	151,685	Human observation	[21]
567	*Urochloa panicoides*	New South Wales/ Australia	-29,033	151,725	Human observation	[21]
568	*Urochloa panicoides*	New South Wales/ Australia	-29,676	150,944	Human observation	[21]
569	*Urochloa panicoides*	Queensland/ Australia	-28,978	151,382	Human observation	[21]
570	*Urochloa panicoides*	New South Wales/ Australia	-29,187	151,368	Human observation	[21]
571	*Urochloa panicoides*	New South Wales/ Australia	-30,631	150,630	Human observation	[21]
572	*Urochloa panicoides*	New South Wales/ Australia	-30,726	150,759	Human observation	[21]
573	*Urochloa panicoides*	New South Wales/ Australia	-31,746	150,671	Human observation	[21]
574	*Urochloa panicoides*	New South Wales/ Australia	-31,668	149,915	Human observation	[21]
575	*Urochloa panicoides*	New South Wales/ Australia	-31,584	149,899	Human observation	[21]
576	*Urochloa panicoides*	New South Wales/ Australia	-30,993	150,378	Human observation	[21]
577	*Urochloa panicoides*	New South Wales/ Australia	-30,972	150,342	Human observation	[21]
578	*Urochloa panicoides*	New South Wales/ Australia	-30,982	150,265	Human observation	[21]
579	*Urochloa panicoides*	New South Wales/ Australia	-30,919	150,186	Human observation	[21]
580	*Urochloa panicoides*	New South Wales/ Australia	-30,739	150,077	Human observation	[21]
581	*Urochloa panicoides*	New South Wales/ Australia	-30,734	150,069	Human observation	[21]
582	*Urochloa panicoides*	New South Wales/ Australia	-30,720	150,055	Human observation	[21]
583	*Urochloa panicoides*	New South Wales/ Australia	-30,618	150,047	Human observation	[21]
584	*Urochloa panicoides*	New South Wales/ Australia	-30,537	150,023	Human observation	[21]
585	*Urochloa panicoides*	New South Wales/ Australia	-30,401	149,892	Human observation	[21]
586	*Urochloa panicoides*	New South Wales/ Australia	-30,254	149,682	Human observation	[21]
587	*Urochloa panicoides*	New South Wales/ Australia	-30,163	149,270	Human observation	[21]
588	*Urochloa panicoides*	New South Wales/ Australia	-29,333	149,134	Human observation	[21]
589	*Urochloa panicoides*	New South Wales/ Australia	-29,279	149,283	Human observation	[21]
590	*Urochloa panicoides*	New South Wales/ Australia	-30,993	150,378	Human observation	[21]
591	*Urochloa panicoides*	New South Wales/ Australia	-30,618	150,047	Human observation	[21]
592	*Urochloa panicoides*	New South Wales/ Australia	-32,173	150,866	Human observation	[21]
593	*Urochloa panicoides*	New South Wales/ Australia	-32,172	150,864	Human observation	[21]
594	*Urochloa panicoides*	New South Wales/ Australia	-32,172	150,863	Human observation	[21]
595	*Urochloa panicoides*	New South Wales/ Australia	-32,173	150,865	Human observation	[21]
596	*Urochloa panicoides*	New South Wales/ Australia	-32,356	150,866	Human observation	[21]
597	*Urochloa panicoides*	New South Wales/ Australia	-32,330	150,858	Human observation	[21]
598	*Urochloa panicoides*	New South Wales/ Australia	-33,581	150,698	Human observation	[21]
599	*Urochloa panicoides*	New South Wales/ Australia	-33,532	150,756	Human observation	[21]

600	*Urochloa panicoides*	New South Wales/ Australia	-35,838	147,209	Human observation	[21]
601	*Urochloa panicoides*	New South Wales/ Australia	-30,656	150,191	Human observation	[21]
602	*Urochloa panicoides*	New South Wales/ Australia	-30,634	150,193	Human observation	[21]
603	*Urochloa panicoides*	New South Wales/ Australia	-31,045	148,134	Human observation	[21]
604	*Urochloa panicoides*	New South Wales/ Australia	-34,066	150,741	Human observation	[21]
605	*Urochloa panicoides*	New South Wales/ Australia	-34,080	150,832	Human observation	[21]
606	*Urochloa panicoides*	New South Wales/ Australia	-30,976	150,274	Human observation	[21]
607	*Urochloa panicoides*	New South Wales/ Australia	-31,511	145,885	Human observation	[21]
608	*Urochloa panicoides*	New South Wales/ Australia	-35,074	148,109	Human observation	[21]
609	*Urochloa panicoides*	New South Wales/ Australia	-30,995	148,321	Human observation	[21]
610	*Urochloa panicoides*	New South Wales/ Australia	-29,775	151,431	Human observation	[21]
611	*Urochloa panicoides*	New South Wales/ Australia	-31,668	149,915	Human observation	[21]
612	*Urochloa panicoides*	New South Wales/ Australia	-30,254	149,682	Human observation	[21]
613	*Urochloa panicoides*	New South Wales/ Australia	-30,163	149,270	Human observation	[21]
614	*Urochloa panicoides*	New South Wales/ Australia	-28,664	150,269	Human observation	[21]
615	*Urochloa panicoides*	New South Wales/ Australia	-32,194	150,095	Human observation	[21]
616	*Urochloa panicoides*	New South Wales/ Australia	-33,844	150,795	Human observation	[21]
617	*Urochloa panicoides*	New South Wales/ Australia	-32,172	150,864	Human observation	[21]
618	*Urochloa panicoides*	New South Wales/ Australia	-32,326	150,739	Human observation	[21]
619	*Urochloa panicoides*	New South Wales/ Australia	-31,098	150,861	Human observation	[21]
620	*Urochloa panicoides*	New South Wales/ Australia	-31,135	150,957	Human observation	[21]
621	*Urochloa panicoides*	New South Wales/ Australia	-33,333	151,433	Human observation	[21]
622	*Urochloa panicoides*	New South Wales/ Australia	-30,233	149,904	Human observation	[21]
623	*Urochloa panicoides*	New South Wales/ Australia	-31,126	150,007	Human observation	[21]
624	*Urochloa panicoides*	New South Wales/ Australia	-29,573	150,626	Human observation	[21]
625	*Urochloa panicoides*	New South Wales/ Australia	-32,140	150,370	Human observation	[21]
626	*Urochloa panicoides*	New South Wales/ Australia	-32,280	150,910	Human observation	[21]
627	*Urochloa panicoides*	New South Wales/ Australia	-32,337	150,584	Human observation	[21]
628	*Urochloa panicoides*	New South Wales/ Australia	-33,750	150,866	Human observation	[21]
629	*Urochloa panicoides*	New South Wales/ Australia	-28,833	153,325	Human observation	[21]
630	*Urochloa panicoides*	New South Wales/ Australia	-28,833	153,314	Human observation	[21]
631	*Urochloa panicoides*	New South Wales/ Australia	-32,299	150,701	Human observation	[21]
632	*Urochloa panicoides*	New South Wales/ Australia	-33,872	150,815	Human observation	[21]
633	*Urochloa panicoides*	New South Wales/ Australia	-34,127	150,715	Human observation	[21]
634	*Urochloa panicoides*	New South Wales/ Australia	-34,129	150,711	Human observation	[21]
635	*Urochloa panicoides*	New South Wales/ Australia	-34,121	150,729	Human observation	[21]
636	*Urochloa panicoides*	New South Wales/ Australia	-34,128	150,714	Human observation	[21]
637	*Urochloa panicoides*	New South Wales/ Australia	-34,108	150,736	Human observation	[21]
638	*Urochloa panicoides*	New South Wales/ Australia	-34,114	150,737	Human observation	[21]
639	*Urochloa panicoides*	New South Wales/ Australia	-34,122	150,738	Human observation	[21]
640	*Urochloa panicoides*	New South Wales/ Australia	-34,119	150,738	Human observation	[21]
641	*Urochloa panicoides*	New South Wales/ Australia	-34,122	150,733	Human observation	[21]
642	*Urochloa panicoides*	New South Wales/ Australia	-34,110	150,735	Human observation	[21]

643	*Urochloa panicoides*	New South Wales/ Australia	-34,117	150,731	Human observation	[21]
644	*Urochloa panicoides*	New South Wales/ Australia	-34,119	150,730	Human observation	[21]
645	*Urochloa panicoides*	New South Wales/ Australia	-34,079	150,736	Human observation	[21]
646	*Urochloa panicoides*	New South Wales/ Australia	-34,112	150,736	Human observation	[21]
647	*Urochloa panicoides*	New South Wales/ Australia	-34,093	150,736	Human observation	[21]
648	*Urochloa panicoides*	New South Wales/ Australia	-34,093	150,735	Human observation	[21]
649	*Urochloa panicoides*	New South Wales/ Australia	-34,081	150,739	Human observation	[21]
650	*Urochloa panicoides*	New South Wales/ Australia	-30,731	150,514	Human observation	[21]
651	*Urochloa panicoides*	New South Wales/ Australia	-31,584	149,899	Human observation	[21]
652	*Urochloa panicoides*	New South Wales/ Australia	-32,073	150,110	Human observation	[21]
653	*Urochloa panicoides*	New South Wales/ Australia	-32,435	151,050	Human observation	[21]
654	*Urochloa panicoides*	New South Wales/ Australia	-30,151	145,863	Human observation	[21]
655	*Urochloa panicoides*	Belgium	50,921	3,139	Human observation	[21]
656	*Urochloa panicoides*	Belgium	50,939	3,138	Human observation	[21]
657	*Urochloa panicoides*	Belgium	50,986	3,422	Human observation	[21]
658	*Urochloa panicoides*	Belgium	51,142	3,790	Human observation	[21]
659	*Urochloa panicoides*	Belgium	51,106	3,748	Human observation	[21]
660	*Urochloa panicoides*	Ethiopia	5,124	44,048	Human observation	[21]
661	*Urochloa panicoides*	Queensland/ Australia	-27,639	152,042	Human observation	[21]
662	*Urochloa panicoides*	Queensland/ Australia	-25,665	150,834	Human observation	[21]
663	*Urochloa panicoides*	Queensland/ Australia	-24,466	148,629	Occurrence	[21]
664	*Urochloa panicoides*	Yunnan/ China	24,817	98,888	Occurrence	[21]
665	*Urochloa panicoides*	Kenya	-2,000	40,000	Occurrence	[21]
666	*Urochloa panicoides*	Kenya	-3,000	39,000	Occurrence	[21]
667	*Urochloa panicoides*	Kenya	-4,000	39,000	Occurrence	[21]
668	*Urochloa panicoides*	Botswana	-22,000	24,000	Occurrence	[21]
669	*Urochloa panicoides*	Eritrea	15,000	39,000	Occurrence	[21]
670	*Urochloa panicoides*	Eswatini	-26,500	31,500	Present	[20]
671	*Urochloa panicoides*	Ethiopia	9,000	39,500	Present	[20]
672	*Urochloa panicoides*	Kenya	1,000	38,000	Present	[20]
673	*Urochloa panicoides*	Malawi	-13,500	34,000	Present	[20]
674	*Urochloa panicoides*	Mauritius	-20,300	5,758	Present	[20]
675	*Urochloa panicoides*	Mozambique	-18,250	35,000	Present	[20]
676	*Urochloa panicoides*	Namibia	-22,000	17,000	Present	[20]
677	*Urochloa panicoides*	Somalia	6,000	48,000	Present	[20]
678	*Urochloa panicoides*	Sudan	16,000	30,000	Present	[20]
679	*Urochloa panicoides*	Tanzania	-6,000	35,000	Present	[20]
680	*Urochloa panicoides*	Uganda	1,250	32,500	Present	[20]
681	*Urochloa panicoides*	Zambia	-1,433	28,500	Present	[20]
682	*Urochloa panicoides*	Zimbabwe	-19,000	29,750	Present	[20]
683	*Urochloa panicoides*	Bhutan	27,500	90,500	Present	[20]
684	*Urochloa panicoides*	India	22,000	79,000	Present	[20]
685	*Urochloa panicoides*	Pakistan	30,000	70,000	Present	[20]

686	*Urochloa panicoides*	Thailand	15,500	101,000	Present	[20]
687	*Urochloa panicoides*	Yemen	15,500	47,500	Present	[20]
688	*Urochloa panicoides*	Ceará/Brazil	-53,213	-3,934	Literature researched	[60]
689	*Urochloa panicoides*	Pernambuco/Brazil	-664,376	-36,868	Literature researched	[60]
690	*Urochloa panicoides*	Córdoba/Argentina	-313,995	-64,194	Literature researched	[13]
691	*Urochloa panicoides*	Tucumán /Argentina	-267,333	-648,334	Literature researched	[61]
692	*Urochloa panicoides*	Córdoba/Argentina	-3,269	-64,722	Literature researched	[62]
693	*Urochloa panicoides*	Tucumán /Argentina	-2,719	-653,097	Literature researched	[63]
694	*Urochloa panicoides*	Santiago del Estero/Argentina	-278,017	-642,670	Literature researched	[39]
695	*Urochloa panicoides*	El Guayacán/Argentina	-266,809	-618,174	Literature researched	[39]
696	*Urochloa panicoides*	Auckland/Nova Zelândia	-3,686	1,747	Literature researched	[64]
697	*Urochloa panicoides*	Mexico	235,541	-1,026	Literature researched	[18]
698	*Urochloa panicoides*	Guaniguanico/Cuba	225,833	-838,331	Literature researched	[65]
699	*Urochloa panicoides*	Cameron/USA	308,643	-969,783	Literature researched	[66]
700	*Urochloa panicoides*	Novo Mexico/USA	34,166	-1,060	Literature researched	[66]
701	*Urochloa panicoides*	Hidalgo county/USA	264,097	-982,241	Literature researched	[66]
702	*Urochloa panicoides*	Zapata/USA	269,093	-9,926	Literature researched	[66]
703	*Urochloa panicoides*	Willacy country/USA	2,646	-9,759	Literature researched	[66]
704	*Urochloa panicoides*	Aguascalientes/Mexico	2,189	-1,023	Literature researched	[67]
705	*Urochloa panicoides*	Jalisco/Mexico	2,084	-1,036	Literature researched	[67]
706	*Urochloa panicoides*	Michoacan/Mexico	19,155	-10,190	Literature researched	[67]
707	*Urochloa panicoides*	Jalisco/Mexico	2,136	-1,019	Literature researched	[68]
708	*Urochloa panicoides*	Jalisco/Mexico	192,406	-1,039	Literature researched	[69]
709	*Urochloa panicoides*	Sierra La Púrica/Mexico	3,057	-109,750	Literature researched	[70]
710	*Urochloa panicoides*	Sierra Madre Occidental /Mexico	259,561	-107,048	Literature researched	[71]
711	*Urochloa panicoides*	Zacatecas/Mexico	227,293	-1,027	Literature researched	[71]
712	*Urochloa panicoides*	Zicuirán-Infiernillo/Mexico	18,847	-1,020	Literature researched	[72]
713	*Urochloa panicoides*	Kobo/Ethiopia	1,215	3,963	Literature researched	[73]
714	*Urochloa panicoides*	Khyber Pakhtunkhwa/Pakistan	3,399	7,168	Literature researched	[74]
715	*Urochloa panicoides*	Gaboroni/Botswana	-246,093	2,593	Literature researched	[75]
716	*Urochloa panicoides*	Metema/Ethiopia	1,295	3,616	Literature researched	[76]
717	*Urochloa panicoides*	Andhra Pradesh/India	1,588	807,770	Literature researched	[77]
718	*Urochloa panicoides*	Karnataka/India	1,502	763,178	Literature researched	[77]
719	*Urochloa panicoides*	Maharashtra/India	1,882	7,678	Literature researched	[77]
720	*Urochloa panicoides*	Rajasthan/India	2,663	7,387	Literature researched	[77]
721	*Urochloa panicoides*	Moradabad/India	2,884	787,676	Literature researched	[78]
722	*Urochloa panicoides*	Khok Bung/Thailand	172,761	1,018	Literature researched	[79]
723	*Urochloa panicoides*	Pantanal/Brazil	-19,523	-571,005	Literature researched	[19]
724	*Urochloa panicoides*	Kaghan Valley/Pakistan	345,417	73,350	Literature researched	[80]
725	*Urochloa panicoides*	Ecuador	-2,122	-7,834	Literature researched	[81]
726	*Urochloa panicoides*	Kilimanjaro/Thailand	-30,674	373,556	Literature researched	[82]
727	*Urochloa panicoides*	Cordilheira do Sal/Paquistão	325,980	723,701	Literature researched	[74]

728	*Urochloa panicoides*	Mogonye/Botswana	-24,861	256,858	Literature researched	[83]
729	*Urochloa panicoides*	Biswanath Chariali/India	2,672	931,402	Literature researched	[84]
730	*Urochloa panicoides*	Índia	97,732	776,601	Literature researched	[85]

Complementary references

60. Flora do Brasil. *Urochloa panicoides* P. Beauv. Available online: http://reflora.jbrj.gov.br/reflora/listaBrasil/FichaPublicaTaxonUC/FichaPublicaTaxonUC.do?id=FB135135 (access on 01 November 2021).
61. Varela, A.E.; Cabrera, D.C.; De La Veja, M.; De La Fuente, E.B. Dinámica de emergencia de plántulas y control de *Urochloa panicoides* P. Beauv resistente a glifosato en Tucumán (Argentina). *Rev. Agron. Noroeste Argent.* **2011**, *41*, 27–38.
62. Dellafiore, C.M.; Brignone, E.; Scilingo, V. Rol Ecológico Del Zorro Pampeano (*Lycalopex gymnocercus*) Como Dispersor De Semillas En Ambientes De Agroecosistema Y Bosques Serranos De Argentina. *Eur. J. Sci.* **2020**, *16*, 223–233. https://doi.org/10.19044/esj.2020.v16n36p223.
63. Olea, I.; Sabaté, S.; Vinciguerra, F.; Devani, L. Pautas para el manejo de malezas en cultivos extensivos en el Noroeste Argentino. *Avanço Agroind.* **2014**, *35*, 25–34.
64. Edgar, E.; Shand, J.E. Checklist of Panicoid grasses naturalised in New Zealand; with a key to native and naturalised genera and species. *N. Z. J. Bot.* **1987**, *25*, 343–353. https://doi.org/10.1080/0028825X.1987.10413351.
65. Naples, N.E.R.; Oviedo, I.B.; Cruz, R.E. Diversidad florística de la Cordillera de Guaniguanico, Cuba/Floristic diversity of the Cordillera de Guaniguanico, Cuba. *Acta Botánica Cubana* **2018**, *217*, 1–32.
66. Wipff, J.K.; Lonard, R.I.; Jones, S.D.; Hatch, S.L. The genus *Urochloa* (poaceae: Paniceae) in Texas, including one previously unreported species for the state. *SIDA Contrib. Bot.* **1993**, *15*, 405–413.
67. Dávila, P.; Mejia-Saulés, M.; Soriano-Martínez, A.M.; Herrera-Arrieta, Y. Conocimiento taxonómico de la familia Poaceae en México. *Bot. Sci.* **2018**, *96*, 462–514.
68. Morrone, O.; Zuloaga, F.O. Sinopsis del género *Urochloa* (Poaceae: Panicoideae: Paniceae) para México y América Central. *Darwiniana* **1993**, *32*, 59–75.
69. Rivera, L.M.M.; Ortiz-Arrona, C.I. *Investigación y Gestión de los Recursos Naturales de la Cuenca del Rio Ayuquila-Armería, Jalisco*; Universidad de Guadalajara: Guadalajara, México, 2020.
70. Sánchez-Escalante, J.J.; Van Devender, T.R.; Reina-Guerrero, A.L. Preliminary Flora of the Sierra La Púrica, Sonora, Mexico. Available online: https://www.researchgate.net/profile/Jose-Sanchez-Escalante/publication/329761058_Preliminary_Flora_of_the_Sierra_La_Purica_Sonora_Mexico/links/5c197064a6fdccfc70586e81/Preliminary-Flora-of-the-Sierra-La-Purica-Sonora-Mexico.pdf (accessed on 30 June 2022).
71. Arrieta, Y.H.; Ortiz, A.C. Diversidad y distribución de las gramíneas (poaceae) en el estado de Zacatecas. *J. Bot. Res. Inst. Tex.* **2009**, *3*, 775–792.
72. Steinmann, V.W. Flora y Vegetación de la Reserva de la Biosfera Zicuirán-Infiernillo, Michoacán, México. *Bot. Sci.* **2021**, *99*, 661–707. https://doi.org/10.17129/botsci.2706.
73. Nigatu, L.; Hassen, A.; Sharma, J.; Adkins, S.W. Impact of *Parthenium hysterophorus* on grazing land communities in north-eastern Ethiopia. *Weed Biol. Manag.* **2010**, *10*, 143–152. https://doi.org/10.1111/j.1445-6664.2010.00378.x.
74. Ahmad, Z.; Khan, S.M.; Ali, S.; Rahman, I.U.; Ara, H.; Noreen, I.; Khan, A. Indicator species analyses of weed communities of maize crop in district Mardan, Pakistan. *Pak. J. Weed Sci. Res.* **2016**, *22*, 227–238.
75. Ernst, W.H.O.; Veenendaal, E.M.; Kebakile, M.M. Possibilities for dispersal in annual and perennial grasses in a savanna in Botswana. *Vegetatio* **1992**, *102*, 1–11. https://doi.org/10.1007/BF00031700.
76. Sintayehu, A. Weed flora survey in field crops of Northwestern Ethiopia. *Afri. J. Agric. Res.* **2019**, *14*, 749–758. https://doi.org/10.5897/AJAR2019.13947.
77. Sadasivaiah, B.; Priyadarshini, P.; Rao, B.R.P. Three rare grasses (*Eragrostis ciliaris* var. clarkei, *E. tenella* var. insularis and *Urochloa panicoides* var. velutina) from Andhra Pradesh, India. *J. Sci. Trans. Eviron. Technov.* **2013**, *6*, 166–167.
78. Kiran, M.; Rahees, N.; Vishal, V.; Vidyasagaran, K. Floristic diversity and structural dynamics of mangroves in the north west coast of kerala, India. *J. Plant Dev.* **2015**, *7*, 549–553.
79. Kabir, M.E.; Webb, E.L. Community in Conservation: A Case from a Deciduous Forest under Community Management in Northeast Thailand. In Proceedings of the FORTROP II: Tropical Forestry Change in a Changing World, Bangkok, Thailand, 17–20 November 2008.
80. Rahman, I.U.; Afzal, A.; Iqbal, Z.; Ijaz, F.; Ali, N.; Asif, M.; Alam, J.; Majid, A.; Hart, R.; Bussmann, R.W. First insights into the floristic diversity, biological spectra and phenology of Manoor Valley, Pakistan. *Pak. J. Bot* **2018**, *50*, 1113–1124.
81. Labrada, R. Revisión de la lista de malezas cuarentenarias de Ecuador. *Technique* **2015**, *15*, 58–68.

82. Hemp, A. Ecology of the pteridophytes on the southern slopes of Mt. Kilimanjaro. Part II: Habitat selection. *Plant Biol.* **2001**, *3*, 493–523. https://doi.org/10.1055/s-2001-17729.
83. Dangerfield, J.M.; Veenendaal, E.M.; Riddoch, B.J.; Black, H. Termites and land use in south-east Botswana: Variety and abundance of termite surface features. *Botsw. Notes Rec.* **1992**, *24*, 165–179.
84. Kumar, K.K.; Babu, P.K. Diversity of Weed Flora in Citrus at ICAR-Regional Research Centre for Citrus, Biswanath Chariali, Assam, India. *Int. J. Curr. Microbiol. App. Sci.* **2019**, *8*, 2100–2107.
85. Velayudham, L.; Jeyaprakash, K.; Balachandran, N.; Rajantheran, M. *Lindernia micrantha* D. Don (Linderniaceae): A red listed plant species new discovery to Tamil Nadu, India. *Plant Arch.* **2020**, *20*, 1404–1408.
